# Modulation of Nutritional and Biochemical Properties of Wheat Grains Infected by Blast Fungus *Magnaporthe oryzae Triticum* Pathotype

**DOI:** 10.3389/fmicb.2020.01174

**Published:** 2020-06-24

**Authors:** Musrat Zahan Surovy, Nur Uddin Mahmud, Pallab Bhattacharjee, Md. Shaid Hossain, Md. Shabab Mehebub, Mosaddiqur Rahman, Bhaskar Chandra Majumdar, Dipali Rani Gupta, Tofazzal Islam

**Affiliations:** ^1^Institute of Biotechnology and Genetic Engineering (IBGE), Bangabandhu Sheikh Mujibur Rahman Agricultural University, Gazipur, Bangladesh; ^2^Department of Fisheries Technology, Bangabandhu Sheikh Mujibur Rahman Agricultural University, Gazipur, Bangladesh

**Keywords:** disease severity, grain mineral content, BARI Gom 24, protein content, antioxidant activity

## Abstract

Wheat blast disease caused by the *Magnaporthe oryzae Triticum* (*MoT*) pathotype exerts a significant impact on grain development, yield, and quality of the wheat. The aim of this study was to investigate morphological, physiological, biochemical, and nutritional properties of wheat cv. BARI Gom 24 under varying levels of blast disease severity in wheat spikes. Grain morphology, volume, weight, and germination of the infected grains were significantly affected by *MoT*. Biochemical traits specifically grain N, Ca, Mg, and Fe content significantly increased (up to threefold; *p* > 0.05), but organic carbon, Cu, Zn, B, and S content in wheat grains significantly decreased with increased severity of *MoT* infection. The grain crude protein content was about twofold higher (up to 18.5% in grain) in severely blast-infected grains compared to the uninfected wheat (9.7%). Analysis of other nutritional properties such as secondary metabolites revealed that total antioxidant activity, flavonoid, and carotenoid concentrations remarkably decreased (up to threefold) with increasing severity of blast infestation in wheat grain. Grain moisture, lipid, and ash content were slightly increased with the increase in blast severity. However, grain K and total phenolic concentration were increased at a certain level of blast infestation and then reduced with increase in *MoT* infestation.

## Introduction

Wheat is the most important cereal crop in the world due to its widespread distribution and extensive use as food products (Barak et al., 2013). The global production of wheat is more than 700 million tons (Kumar et al., 2017), and it is the second most important cereal crop in Bangladesh after rice. Changes in dietary food habits and the regular uptake of wheat products were increasing in Bangladesh. Wheat is a good source of calories, minerals, proteins, and dietary fibers helping in the prevention and treatment of some digestive disorders. The major components of wheat kernels are bran (13–17%), germ (2–3%), and endosperm (81–84%). Starch (60–75%), proteins (6–20%), moisture (∼10%), and lipids (1.5–2%) are the major constituents of the endosperm (Barak et al., 2013). The grain protein content is an important factor determining the quality of pasta and bread making and also human nutrition. It is an important trait for growers because premium prices are frequently paid on the basis of high grain protein content (Olmos et al., 2003). Grain lipids are distributed unevenly throughout the different parts of the wheat grain (Chung et al., 2009; Day, 2016), and the highest concentration is present in the germ (Uthayakumaran and Wrigley, 2017). The mineral contents (N, P, K, Fe, S, Zn, etc.) of wheat grain also determine the quality of grain (Urashima et al., 2009). The antioxidant profile has been representing a key parameter to predict the shelf life of the product and also protects plants from oxidative damage (Shahidi and Liyana-Pathirana, 2008). Phenolic acids, flavonoids, lignans, carotenoids, tocopherols, and phytosterols are also present in wheat grain and exert beneficial effects on human health (Tsao, 2008). Phenolic compounds are involved in plant defense mechanisms by inducing a barrier to invade phytopathogens. These compounds have antioxidative, anti-inflammatory, antimutagenic, and anticarcinogenic properties to modulate enzymatic functions in the plant cell (Ho et al., 1992; Shahidi and Liyana-Pathirana, 2008). Phenolics are concentrated in the outer layers, pericarp, aleurone, germ, and less in the endosperm of wheat grain (Maillard and Berset, 1995; Atanasova-Penichon et al., 2016). It also neutralizes free radicals, decomposing peroxides, and quenching singlet oxygen (Valifard et al., 2014). Synthesis and accumulation of phenolic compounds are stimulated in the response of pathogenic attack. Flavonoids are present in the leaf tissue of wheat in diversified form, but in grains, they are not diverse in nature (Cavaliere et al., 2005; Asenstorfer et al., 2006). Carotenoids are another group of phytochemicals contributing to pigments and play important roles in the human diet.

Biotic and abiotic stresses are major concerns for wheat production. Fungal diseases have been increasing and limit wheat production worldwide (Fisher et al., 2012). *Magnaporthe oryzae Triticum* (*MoT*) pathotype causes wheat blast disease and can limit wheat production up to 100% (Islam et al., 2016, 2019; Ceresini et al., 2018). It was first reported in the State of Paraná of Brazil in 1985 (Iragashi et al., 1986). Since then, it has become a major constraint to wheat-growing areas in Brazil (Lima, 2004), Argentina (Perelló et al., 2015), Bolivia (Barea and Toledo, 1996), and Paraguay (Viedma et al., 2010). In February 2016, wheat blast disease was first spotted in Bangladesh, which devastated 15,000 hectares of wheat field of eight wheat-growing districts (Meherpur, Chuadanga, Jessore, Barishal, Bhola, Jhenaidah, Pabna, and Kustia) with yield losses up to 100% (Islam et al., 2016). It was spread to 12 other wheat-growing districts (Magura, Faridpur, Rajshahi, Tangail, Comilla, Jamalpur, Natore, Rajbari, Norail, Noagoan, Mymensingh, and Madaripur) in the subsequent years. Meherpur district is known as a hot spot for wheat blast infection in Bangladesh. In 2015, the estimated wheat production was 45,383 metric tons in Meherpur. Owing to *MoT* infestation, it was dramatically reduced into 19,893 metric tons (BBS, 2017) in 2016 and caused 56.17% yield loss. The sudden emergence of wheat blast disease is posing a serious threat to food and nutritional security of Bangladesh and South Asia. However, our understanding of the biology of *MoT* and its interactions with wheat plant is very limited. Better understanding of interactions between *MoT* and wheat plant would help us to design an effective management strategy against the wheat blast disease.

Infection of *MoT* at the grain filling stage results in small, shriveled, light in weight, and discolored grains (Miranda et al., 2015; Islam et al., 2016, 2019). It also affects physiological processes resulting in reduced growth, yield, and nutritional qualities of the grains (Arfan et al., 2007; Urashima et al., 2009). Enhanced knowledge on biochemical and nutritional aspects of *MoT*-infected grains will be helpful in determining the nutritional value of wheat grains and also provides information related to detrimental damaged levels for human consumption.

To date, there is scant information available to describe the nutritional and biochemical changes occurring in wheat grains during *MoT* infection (Urashima et al., 2009; Miranda et al., 2015; Martínez et al., 2019). Infection of wheat grains by the notorious *MoT* pathogen may change the metabolic processes, reduce grain productivity, and also reduce the beneficial dietary contents (Urashima et al., 2009; Martínez et al., 2019). However, to fill the knowledge gaps, the present study was undertaken to: (i) evaluate physical, physiological properties, and mineral contents in grains affected by varying levels of blast disease infestation and (ii) analyze biochemical properties, nutritional contents, and antioxidant activities in varying levels of wheat blast damaged grains.

## Materials and Methods

### Sample Collection

Wheat cv. BARI Gom 24 was cultivated in the majority of wheat-growing areas of Meherpur in 2016. High yield, big spike, large grain, and lodging tolerance are the main attractive attributes of BARI Gom 24 cultivar (Pandit et al., 2011; Rashid and Hossain, 2016). Similarly, BARI Gom 26 is another high yielding and superior wheat variety in Bangladesh. In February 2016, severely affected BARI Gom 24 and BARI Gom 26 cultivated in farmer’s fields were randomly selected. The seed sowing was performed by broadcasting method in both varieties.

**FIGURE 1:**
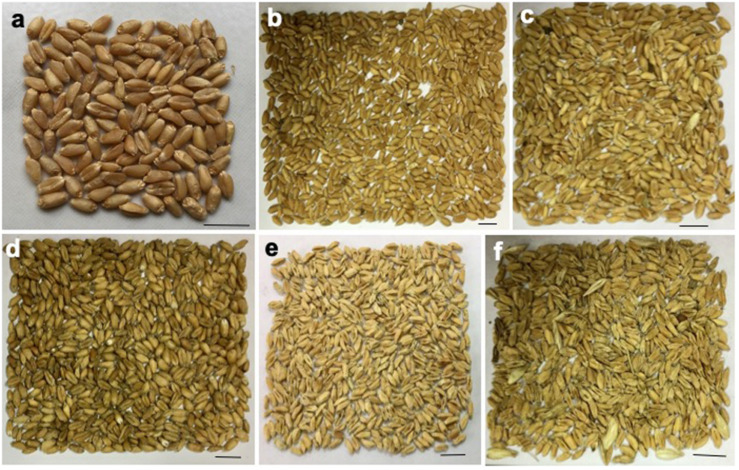
Morphological features of intact grains of blast affected and unaffected wheat cv. BARI Gom 24 spike. **(a)** Category 1: unaffected (control). **(b)** Category 2: 1–19% damaged. **(c)** Category 3: 30–39% damaged. **(d)** Category 4: 40–59% damaged. **(e)** Category 5: 60–79% damaged. **(f)** Category 6: 80–99% damaged wheat grains.

**FIGURE 2:**
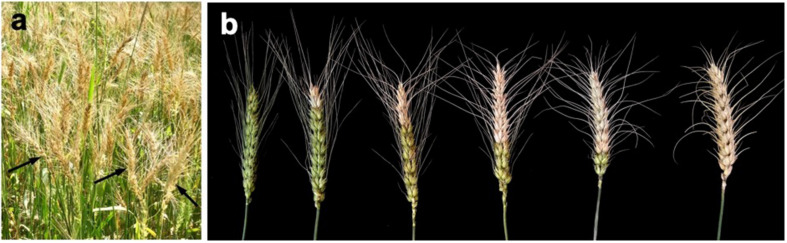
Wheat blast symptoms in the field and spikes. **(a)** Almost 100% bleached spikes (arrows) of a severely blast-infected wheat cv. BARI Gom 24 in the field in Meherpur district of Bangladesh. **(b)** Characteristic wheat blast disease symptom in wheat spikes (head blast) of varying levels of severity (from left to right), category of damage 1–6. Upper part of the point of infection of the spikes became bleached due to blockage and/or blocking of vascular system of the rachis by the mycelial growth of *Magnaporthe oryzae Triticum* (*MoT*).

Complete or partial bleached spikes above the point of infection with no grain or shriveled grain were commonly found in all affected heads by the *MoT* (Figures 1, 2). The field symptoms of wheat blast are shown in Figure 2. The wheat spikes (300 spikes per category) were collected by cutting the ear from the wheat plants. Visual observation of the wheat spikes was used to categorize the disease severity (Figures 1, 2). A simple modification of the study of Murakami et al. (2000), wheat spikes were categorized into six different categories: category 1 (no visible symptom on the spike and the grains); category 2 (1–19% bleached spike and shriveled grains); category 3 (20–39% bleached spike and shriveled grains); category 4 (40–59% bleached spike and shriveled grains); category 5 (60–79% bleached spike and shriveled grains); and category 6 (≥80% bleached spike and shriveled grains). However, only three categories (1, 3, and 4) of the damaged wheat grains were obtained for BARI Gom 26 due to unavailability of other categories in that field for this variety.

Exactly 300 spikes were collected from the field for each category for the experimental purposes, and all grains were separated from the spikes. Grains were stored at 4°C in a zipped lock polybag until further studies.

### Physical Observations of Infected Grains

Based on the preliminary visual observations, around 250 g of grains were used from each category of infected spikes and ca. 1,000 grains were selected randomly for evaluation of physical parameters (grain length, breadth, 1,000 grain weight, germination, and volume of 500 grains). The breadth and lengths of the grains were measured using a slide caliper. The weight of 1,000 grains was recorded using a digital weight machine. For germination test, 25 sterilized seeds were placed in a Petri dish containing water-soaked filter paper. Germination percentage was recorded after 7 days and calculated using the following formula:

$$\mathrm{Germination}\,\mathrm{percentage}\;=\left(\frac{\mathrm{Number}\,\mathrm{of}\,\mathrm{seeds}\,\mathrm{germinated}}{\mathrm{Total}\,\mathrm{number}\,\mathrm{of}\,\mathrm{seeds}\,\mathrm{set}\,\mathrm{for}\,\mathrm{germination}}\right)\times 100\%$$

### Estimation of Mineral Contents

Dried wheat grain samples were ground finely in an electric grinder for mineral analysis. The nitrogen (N) percentage (%N) was determined through the micro-Kjeldahl method (Jackson, 1973). Phosphorus (P) content was determined by colorimetric method (Page et al., 1982) and the potassium (K) content by the flame emission spectrophotometric method (Ghosh et al., 1983). Grain sulfur (S) and calcium (Ca) contents were determined by turbidimetric (Tandon, 1995) and complexometric method (Page et al., 1982), respectively. Magnesium (Mg), boron (B), and zinc (Zn) contents were analyzed by Azomethine-H and atomic absorption spectrophotometric method as described by Page et al. (1982).

### Estimation of Protein, Moisture, Lipid, and Ash Contents in Wheat Grains

The contents of crude protein, moisture, lipid, and ash in wheat grains were determined by using standard AOAC methods (AOAC, 2000).

#### Crude Protein Percentage

Crude protein was determined using the micro-Kjeldahl method. The percentage of crude protein in the sample was calculated as:

Nitrogen as % crude protein = % N × F, where N is nitrogen and F (conversion factor) is equivalent to 5.7 (Martínez et al., 2019).

#### Moisture Content

The moisture content of the grain was determined by weighing 2.0 g of sample into a pre-weighed china dish and drying it in an air-forced draft oven at a temperature of 105 ± 5°C until the constant weight of dry matter was obtained. The moisture content in the grain was determined as follows:

%Moisture =[{(Weightoforiginalsample-Weightofdriedsample)/Weightoforiginalsample}×100%](AOAC, 2000)

#### Lipid Content

Two grams of grain samples were weighed in triplicate. The samples were extracted with 200 ml of petroleum ether for 6 h. The solvent-free fat in the flux was dried in an oven at 105°C for an hour and cooled in desiccators. The final weight was recorded, and the lipid content was determined as follows:

%Lipid=(Weightofextract/Weightofsample)×100%(AOAC, 2000).

#### Ash Content

Two grams of grain samples were incinerated in a muffle furnace at 550°C for 4 h and cooled in a desiccator. The dried sample was weighed and powdered. The final weight after powder was also registered. The ash content was determined as follows:

%Ash=(WeightofAsh/Weightofsample)×100%(AOAC, 2000).

### Determination of Total Carotenoids, Flavonoids, and Phenolics of Wheat Grain

Hundred grams of grains were finely ground, and 1.0 g of ground grains was extracted in 40 ml of 90% aqueous methanol in a tightly capped bottle. The mixture was homogenized by a standard homogenizer for 1 h. The extract was filtered, and sub-samples were used for the determination of total flavonoids, total phenolics, and total antioxidant capacity. To determine carotenoid concentration, 5.0 ml of acetone was added to 2 g of homogenized seed sample in a glass vial. Then, it was incubated for 24 h in the dark at 4°C. Three milliliters of supernatant was taken in a glass cuvette, and the absorbance of the acetone extract was measured at 444 nm using acetone as blank in the spectrophotometer (PD-303UV Apel spectrophotometer, United States) mentioned above in triplicate. Total carotenoid concentration was measured in mg per g of a sample as lutein equivalent according to the protocol described earlier (Rahman et al., 2018). The AlCl_3_ colorimetric method was used to determine the total flavonoid concentration (TFC) of wheat grain extract. In a test tube, 1.0 ml of methanol extract of seed sample was added to 0.4 ml of 5% sodium nitrate, 0.6 ml of 10% AlCl_3_.6H_2_O (5 min later) at room temperature. Five minutes later, 2.0 ml of 1 M NaOH was added to the mixture and shaken thoroughly. For blank reaction, 1.0 ml of methanol was taken instead of methanol extract of grain samples. A PD-303UV Apel spectrophotometer (United States) was used to take the absorbance of the solution reaction mixture at 510 nm against the blank sample (Zhishen et al., 1999). The measurements were compared to a standard curve of quercetin solutions, and TFC was expressed as μg/g FW quercetin equivalent. Total phenolic compound concentration was determined by the Folin–Ciocalteau method. In a test tube, ca. 0.5 ml of 10% (0.2 N) Folin–Ciocalteau reagent was added with 1.0 ml of methanol extract of wheat seed sample and 1.0 ml of methanol alone as blank. The test tubes were shaken for 10 s, covered, and incubated for 15 min at room temperature. Aqueous 700 mM sodium carbonate (Na_2_CO_3_) solution (2.5 ml) was added to each reaction mixture, then vortexed, covered, and incubated at room temperature (25°C) for 2 h. The absorbance of the solution was measured at 765 nm against the blank sample (Ainsworth and Gillespie, 2007). The measurements were compared to a standard curve of gallic acid solutions, and total phenolics were expressed as μg/g FW gallic acid equivalent (Rahman et al., 2018).

### Antioxidant Activity

1,1-Diphenyl-2-picrylhydrazyl (DPPH; CalBiochem, Germany) radical scavenging assay (Kitts et al., 2000; Rahman et al., 2018) was used to estimate the antioxidant activity. In a test tube, 1.0 ml of DPPH solution (0.0788 g of 0.2 mM DPPH in 1 L of methanol) was added to 1.0 ml of methanol extracted supernatant of grain sample for 5 min at 25°C. A PD-303UV Apel spectrophotometer (United States) was used to read the absorbance at 517 nm. When the antioxidants react with DPPH, the DPPH is reduced to DPPH-H, and as a consequence, the absorbance decreases. The DPPH-H formation results in decolorization (yellow color) concerning the number of electrons captured (Kitts et al., 2000). The DPPH solution with corresponding solvents (i.e., without plant material) served as the control. Methanol with the respective plant extracts was used as the blank. The DPPH radical scavenging activity of grain extracts was calculated as the percentage inhibition (Rahman et al., 2018).

%InhibitionofDPPHradicalactivity =(A⁢Control-A⁢Sample)/A⁢Control×100%

### Statistical Analysis

The data were statistically analyzed using SPSS version 16 and Microsoft Excel 2016 software. Fisher’s least significant difference (LSD) was performed to determine the significant difference between means at a significance level of *p* ≤ 0.05 and reported as the mean ± standard deviation (SD). Each experiment was repeated thrice with nearly identical results.

## Results

### Evaluation of Physical Parameters and Germination of *MoT*-Infected Grains

#### Grain Length and Breadth

Grain breadth and length differed significantly (*p* ≤ 0.05) at varying levels of *MoT* infestation in wheat grains compared to uninfected healthy grains (Table 1). On average, seed length ranged from 4.86 to 6.96 mm. The maximum grain length was recorded in category 1 (no infection) followed by category 2 (1–19% damaged) grains, and the values were 6.96 and 6.90 mm, respectively. Average grain length was highly affected in category 6 (≥80% damaged) grains (4.86 mm). The grain breadth also showed a significant drop with an increase in *MoT* infestation. The maximum grain breadth was recorded in unaffected healthy category 1 (2.80 mm), and the minimum was found in category 6 (≥80% damaged) grains (1.30 mm) (Table 1). There was about twofold variation between highly infested grains and unaffected healthy grains. A significant positive correlation was recorded between grain length and grain breadth. Both grain length and breadth in BARI Gom 26 also reduced due to blast severity (Supplementary Table S1).

**TABLE 1:** Effects of *Magnaporthe oryzae Triticum* (*MoT*) infestation on physical changes and germination percentage of wheat cv. BARI Gom 24.

**Damage category**	**Grain length (mm)**	**Grain breadth (mm)**	**% of germination**	**Volume of 500 grains (mm^3^)**
1 (No infection)	6.96 ± 0.01a	2.80 ± 0.03a	92.92 ± 0.10a	20.07 ± 0.05a
2 (1–19%)	6.90 ± 0.02a	2.70 ± 0.01a	90.86 ± 0.47b	20.03 ± 0.41a
3 (20–39%)	5.83 ± 0.02b	2.28 ± 0.01b	90.48 ± 0.29b	16.00 ± 0.18b
4 (40–59%)	5.65 ± 0.03c	2.09 ± 0.01c	90.84 ± 0.11b	12.5 ± 0.19c
5 (60–79%)	4.98 ± 0.02 d	1.57 ± 0.05d	90.39 ± 0.15b	7.87 ± 0.11d
6 (≥80%)	4.86 ± 0.03e	1.30 ± 0.02e	86.46 ± 0.22c	5.89 ± 0.07e
CV	0.0581	0.0875	0.7980	0.6136

*Mean values within a column followed by the same letter do not differ significantly by Fisher’s protected least significant difference (LSD) test at *p* ≤ 0.05. Data are presented as mean ± SE (*n* = 3).*

#### Thousand Grain Weight, 500 Grain Volume, and Germination Percentage

**FIGURE 3:**
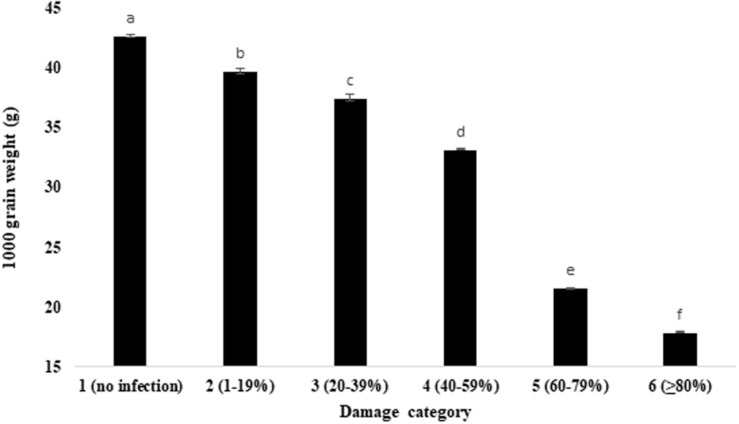
Influence of *MoT* infection in 1,000 grain of wheat cv. BARI Gom 24. Mean values in the bars followed by the same letter(s) are not significantly different as assessed by Fisher’s protected LSD (least significant difference) at *p* ≤ 0.05.

Comparative evaluation of the wheat grain weight and volume under unaffected healthy and blast-damaged grains indicated that the *MoT* infestation caused a significant reduction in 1,000 grain weight (Figure 3) and volume of 500 grains (Table 1). The reduction in 1,000 grain weight was higher in category 6 (17.88 g) grains than those of damaged grains in other categories. The maximum 1,000 grain weight (42.7 g) was found in category 1 (unaffected healthy) grains, whereas the minimum was recorded in category 6 (17.88 g) grains (Figure 3). Similar to 1,000 grain weight, the volume of 500 grains also significantly (*p* ≤ 0.05) varied among the categories of the grains. The maximum grain volume (20.07 mm^3^) of 500 seeds was also found in category 1 (no infection), followed by category 2 (1–19% damaged) grains (20.03 mm^3^). The minimum of 500 grain volume (5.89 mm^3^) is recorded in category 6 (Table 1). There was 2.4- and 3.4-fold reduction in *MoT-*infested grain weight and volume, respectively, compared to unaffected healthy grains. The 1,000 grain weight of BARI Gom 26 was also significantly reduced by blast infestation compared to unaffected healthy wheat grains (Supplementary Table S1). However, blast infestation did not significantly affect the volume of 500 seeds of BARI Gom 26 (Supplementary Table S1).

Grain germination percentage (%) was slightly affected by blast infestation. The germination percentage varied from 86.46 to 92.92%. The maximum germination (92.92%) is registered in category 1 (non-infected healthy) grains, and the minimum is recorded in 80–99% damaged grains of category 6 (86.46%). The blast infestation significantly reduced the germination percentage compared to unaffected healthy grains. Germination percentage in the category 4 seeds of BARI Gom 26 was significantly reduced compared to the unaffected healthy grains.

### *MoT* Infection Changes the Composition of Grain Minerals

Table 2 presents the content of minerals of blast-infected wheat grains. The mineral contents of wheat grains were significantly changed due to *MoT* infestation in wheat grains. The severity of *MoT* infection increased the contents of nitrogen (N), calcium (Ca), magnesium (Mg), and iron (Fe) but decreased organic carbon, phosphorus (P), copper (Cu), zinc (Zn), boron (B), and sulfur (S) contents in wheat grains. However, grain potassium (K) content changed slightly then reduced with increased *MoT* infestation.

**TABLE 2:** Effects of *MoT* infestation on mineral contents of wheat cv. BARI Gom 24.

**Damage categories**	**% N%**	**% OC**	**Ca^2+^ (mg/g)**	**Mg^2+^ (me/100 g)**	**K (me/100g)**	**P (mg/g)**	**Cu (mg/g)**	**Fe (mg/g)**	**Zn (mg/g)**	**B (μg/g)**	**S (μg/g)**
1 (No infection)	1.70 ± 0.10e	4.00 ± 0.36a	0.21 ± 0.02c	0.10 ± 0.01a	1.0 ± 0.005b	3.1 ± 0.02a	4.20 ± 0.27a	53.20 ± 0.50d	52.20 ± 0.23a	52.00 ± 0.41a	0.36 ± 0.02a
2 (1–19%)	2.37 ± 0.02d	3.79 ± 0.12a	0.22 ± 0.01c	0.10 ± 0.003a	1.50 ± 0.02a	3.0 ± 0.005a	4.00 ± 0.03a	54.20 ± 0.49cd	48.43 ± 0.27b	30.00 ± 0.07b	0.33 ± 0.005a
3 (20–39%)	2.78 ± 0.09c	3.59 ± 0.04a	0.25 ± 0.01c	0.11 ± 0.01a	1.50 ± 0.017a	2.8 ± 0.02ab	3.80 ± 0.08ab	54.20 ± 0.25cd	42.00 ± 0.26c	28.00 ± 0.08c	0.28 ± 0.01b
4 (40–59%)	2.92 ± 0.02bc	3.29 ± 0.18a	0.41 ± 0.01b	0.12 ± 0.01a	1.50 ± 0.01a	2.7 ± 0.01ab	3.20 ± 0.10bc	55.20 ± 0.28c	34.87 ± 0.07d	25.00 ± 0.13d	0.26 ± 0.01bc
5 (60–79%)	3.18 ± 0.03ab	3.20 ± 0.13a	0.47 ± 0.02b	0.12 ± 0.02a	1.00 ± 0.12b	2.3 ± 0.02b	2.60 ± 0.14c	73.20 ± 0.41b	14.05 ± 0.11e	20.00 ± 0.11e	0.23 ± 0.006c
6 (≥80%)	3.25 ± 0.02a	2.00 ± 0.02b	0.67 ± 0.03a	0.13 ± 0.005a	1.00 ± 0.03b	2.4 ± 0.005b	1.20 ± 0.06d	89.40 ± 0.22a	12.00 ± 0.03f	17.00 ± 0.26f	0.11 ± 0.01d
CV	0.176	0.555	0.051	0.035	0.1520	0.037	0.4218	1.165	0.570	0.671	0.031

*Mean values within a column followed by the same letter do not differ significantly by Fisher’s protected LSD test at (*p* ≤ 0.05). Data are presented as mean ± SE (*n* = 3). N, nitrogen; OC, organic carbon; Ca, calcium; Mg, magnesium; K, potassium; P, phosphorus; Cu, copper; Fe, iron; Zn, zinc; B, boron; S, sulfur.*

#### Nitrogen (N) and Organic Carbon Contents

In this study, the content of nitrogen (N) varied from 1.70 to 3.25%. High N content (3.25%) was obtained in category 6 (Table 2), which was ca. twofold higher than unaffected control. The range of organic carbon content was 2.00–4.00%. The percentage of organic carbon was simply reversed in trend. High organic carbon (4%) was noted in unaffected control category 1 grains followed by category 2 (3.79%) grains. The minimum value (2.0%) was recorded in category 6 grains. Compared with N content, organic carbon content showed a simply reversed trend. Similar trends of N content and organic carbon were also obtained in blast-damaged grains of BARI Gom 26 (Supplementary Table S2).

#### Calcium and Magnesium Contents

The calcium (Ca) content ranged from 0.21 to 0.67 mg/g. The highest Ca content (0.67 mg/g) was registered in category 6 grains (80–99% damaged), and the lowest in non-infected category 1 grains (0.21 mg/g). The magnesium (Mg) content slightly increased with the increase in disease severity and ranged from 0.10 to 0.13 me/100 g. In category 4 and category 5 damaged grains, the Mg contents were the same (0.12 me/100 g), and the highest content was recorded in category 6 damaged grains (0.13 me/100 g) (Table 2). Although Ca content increased twofold in category 4 compared to healthy wheat grains in BARI Gom 26, Mg content remained unchanged (Supplementary Table S2).

#### Potassium and Phosphorus Contents

In this study, the potassium (K) content varied from 1.0 to 1.50 me/100 g. The K content was increased up to the damage of 40–59% of grains (category 4), and then, the values were decreased with increased disease severity. Like organic carbon percentage, the phosphorus (P) content was decreased when the severity of blast infestation level increased at a certain level (Table 2). The maximum value of P content was found in unaffected grains (category 1; 3.1 mg/g), and it was significantly higher than all other categories. The minimum P content was found in category 5 grains (2.3 mg/g). On the other hand, in BARI Gom 26, both K and P contents were significantly increased with increasing blast infestation (Supplementary Table S2).

#### Copper and Iron Content

Copper (Cu) content showed remarkable variations in terms of damaged levels of the wheat grains (1.20–4.20 mg/g). The highest Cu content (4.20 mg/g) was found in the unaffected category 1 grains, and the lowest (1.20 mg/g) was in category 6 grains. Iron (Fe) content was varied from 53.20 to 89.40 mg/g. The significant and notably higher Fe content (89.40 mg/g) was found in category 6 (more than 80% damaged) grains, and lower (53.20 mg/g) was recorded in unaffected healthy category 1 grains. Almost similar trends of changes in Cu and Fe contents were recorded in BARI Gom 26 due to blast infestation (Supplementary Table S2).

#### Zinc, Boron, and Sulfur Contents

The zinc (Zn) content of wheat grains varied significantly in terms of blast infestation. The higher the blast infestation, the lower the Zn content in grains. It ranged from 12.0 to 52.20 mg/g. High (53.20 mg/g) Zn content was observed in unaffected category 1 grains followed by damaged category 2 (48.43 mg/g) grains. The minimum Zn content (12.00 mg/g) was recorded in heavily damaged category 6 grains (Table 2). It revealed that grain Zn content reduced up to fourfold by the blast infestation.

The boron (B) content was significantly varied from 17 to 52 μg/g (Table 2). The maximum B content (52 μg/g) was obtained in unaffected category 1 grains. The minimum B content (17 μg/g) was found in category 6 grains (Table 2), which is about threefold lower than the content in healthy grains. Similarly, maximum S content was recorded in healthy category 1 grains (0.36 μg/g), and the minimum content (0.11 μg/g) was in category 6 grains (Table 2). Almost similar trends of the contents of Zn, B, and S in blast-affected wheat grains of BARI Gom 26 were also recorded (Supplementary Table S2).

### Changes in Crude Protein, Moisture, Lipid, and Ash Percentage

**FIGURE 4:**
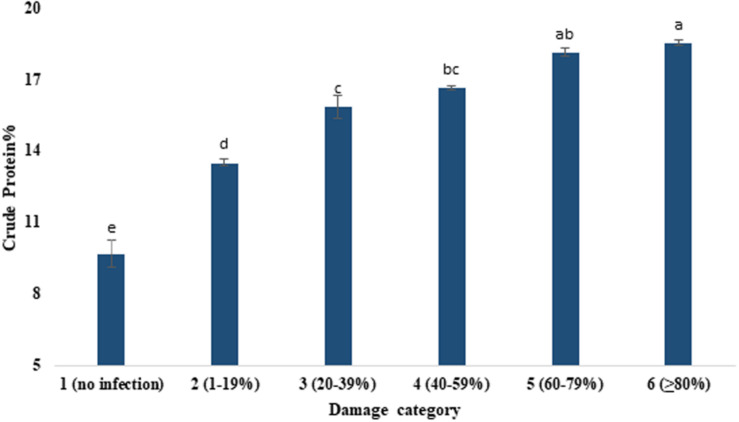
Enhancement of crude protein content (%) by the severity of *MoT* infection in wheat cv. BARI Gom 24. Mean values in the bars followed by the same letter(s) are not significantly different as assessed by Fisher’s protected LSD (least significant difference) at *p* ≤ 0.05.

Table 3 presents the proximate compositions of wheat grains. Crude protein content ranged from 9.69 to 18.53%. It was increased with the increased severity of *MoT* infestation (Figure 4). Category 6 grains (18.53%) had higher protein content followed by the grains of category 5 (18.13%). The lowest crude protein percentage (9.69%) was found in unaffected grains of category 1 (Figure 4). The protein content (average) increased nearly twofold due to blast severity compared to unaffected healthy grains. The moisture percentage ranged from 18.36 to 18.73%. The maximum moisture content was recorded in highly damaged grains but did not exhibit pronounced variations among the various levels of blast-affected grains (Table 3).

**TABLE 3:** Effect of *MoT* infestation on moisture, lipid, and ash contents in wheat cv. BARI Gom 24.

**Damage category**	**Moisture %**	**Lipid %**	**Ash %**
1 (No infection)	18.36 ± 0.32a	3.28 ± 0.10a	1.99 ± 0.05a
2 (1–19%)	18.39 ± 0.14a	3.39 ± 0.007a	2.10 ± 0.09a
3 (20–39%)	18.58 ± 0.21a	3.40 ± 0.12a	2.15 ± 0.12a
4 (40–59%)	18.62 ± 0.42a	3.44 ± 0.05a	2.21 ± 0.02a
5 (60–79%)	18.71 ± 0.04a	3.49 ± 0.08a	2.26 ± 0.14a
6 (≥80%)	18.73 ± 0.26a	3.55 ± 0.10a	2.35 ± 0.03a
CV	0.8105	0.2811	0.2716

*Mean values within a column followed by the same letter do not differ significantly by Fisher’s protected LSD test at (*p* ≤ 0.05). Data are presented as mean ± SE (*n* = 3).*

Similar to grain moisture content, lipid and ash contents of the grains were slightly increased with the increase in disease severity (Table 3). The maximum lipid (3.55%) and ash (2.35%) contents were noted in category 6 grains where unaffected healthy grains had the minimum values of these two parameters (Table 3). Similarly, in BARI Gom 26, protein content significantly increased with increasing infestation of the blast (Supplementary Table S3). The other two parameters, grain lipid and ash contents, remained statistically unchanged.

### Modulation of Nutritional Concentrations in Blast-Affected Wheat Grains

#### Total Flavonoids and Phenolics

**FIGURE 5:**
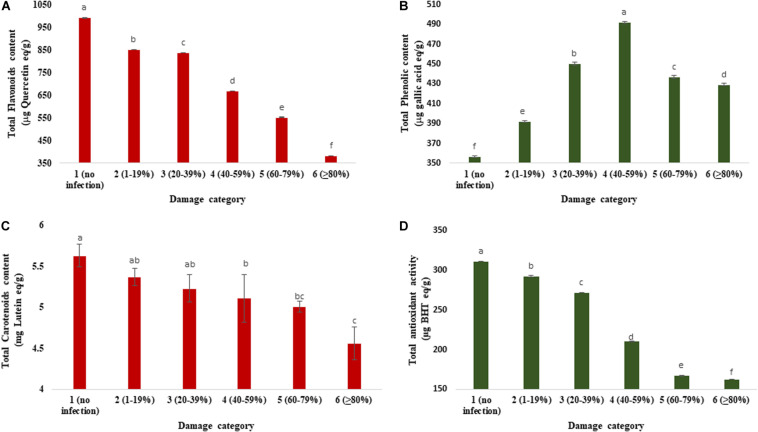
Changes in total flavonoids, total phenolic, total carotenoid concentrations, and antioxidant activity in variously damaged wheat grains cv. BARI Gom 24 infected by *MoT*. **(A)** Reduction in total flavonoid concentration (μg quercetin equivalent/g). **(B)** Changes in total phenolic concentration (μg gallic acid equivalent/g). **(C)** Reduction in total carotenoid concentration in mg lutein equivalent/g). **(D)** Total antioxidant activity (μg BHT equivalent/g). Mean values in the bars followed by the same letter(s) are not significantly different as assessed by Fisher’s protected LSD (least significant difference) at *p* ≤ 0.05.

Figure 5 exhibits the changes in total flavonoids, phenolics, carotenoids, and antioxidants in wheat grains as influenced by varying levels of severity of blast infestation. The range of TFC was 382.37 to 990.68 μg quercetin/g. Category 1 (990.68 μg quercetin/g) had a higher TFC than the other categories of the grains. Category 6 (≥80% damaged) grains had the lowest TFC (382.37 μg quercetin/g), which was about 2.5-fold lower than the unaffected control (Figure 5A). The total phenolic concentration (TPC) significantly varied from 356.93 to 491.70 μg gallic acid/g among the different categories of the wheat grains. Initially, the TPC was increased (up to 40–59% of damaged grains), but after a certain level (60–79% of damaged grains), the TPC was slightly decreased (Figure 5B). The maximum concentration (491.70 μg gallic acid/g) of TPC was estimated in category 4 (40–59% damaged grains), followed by category 3 (450.26 μg gallic acid/g). The minimum concentration (356.93 μg gallic acid/g) was registered in category 1 (no infection) of the healthy wheat grains. In BARI Gom 26, the concentration of total flavonoids decreased with increasing blast infestation; however, a reverse phenomenon was found in the concentration of total phenolics (Supplementary Table S4).

#### Total Carotenoids

Infection by *MoT* in wheat grain significantly decreased total carotenoid concentration (Figure 5C). The total carotenoid concentration in various categories of the grains ranged from 5.63 to 4.56 mg (lutein/g). The highest carotenoid concentration was estimated in the grains of category 1 (no infection; 5.63 lutein/g), followed by category 2 (1–19%) damaged grains (5.37 lutein/g). Total carotenoid concentration in unaffected wheat grain was significantly higher than those of blast-affected wheat grains. The minimum carotenoid concentration was estimated in the grains of category 6 (4.56 mg lutein/g) (Figure 5C). Carotenoid concentration in BARI Gom 26 remained statistically unchanged (Supplementary Table S4).

#### Total Antioxidant Activity

The total antioxidant activity (TAA) of wheat grains was estimated by DPPH assay. The *MoT* infestation also remarkably modulated the antioxidant activity in grains of wheat (Figure 5C). The range of TAA (DPPH) was from 162.82 to 311.22 μg BHT/g. The significant decrease in antioxidant activity (DPPH) in grain was observed when the *MoT* was infestation increased. It was constitutively maximum in the case of category 1 (311.22 μg BHT/g) (Figure 5D), and minimum level was found in category 6 grains (162.82 μg BHT/g). Similar to BARI Gom 24, the antioxidant activity was remarkably decreased with increased blast infestation in BARI Gom 26 (Supplementary Table S4).

## Discussion

In the present study, we demonstrated that *MoT* infection significantly modulated physical, physiological, biochemical, and nutritional properties of wheat grains. Physical properties of the grain such as length, breadth, volume, and 1,000 grain weight significantly decreased with increased severity of the wheat grains by the *MoT* infestation. A significant negative impact of wheat blast disease was recorded in some grain mineral contents such as P, Cu, Zn, B, and S, and organic carbon. Blast infestation significantly increased TPC, N, crude protein, Ca, and Fe contents in wheat grains. However, Mg, K, moisture, ash, and lipid contents were slightly increased with increased *MoT* infestation. Germination of blast-infested grains was considerably decreased compared to the unaffected healthy wheat grains. Changes in some biochemical parameters in blast-affected wheat grains shown in this study are similar to a report published by Urashima et al. (2009). To our knowledge, this is the first report on quantitative estimation of modulation of physical, physiological, biochemical, and nutritional properties of wheat grains affected by the varying levels of *MoT* infestation. We found an almost similar trend of change in the contents of nutritional and biochemical properties of grains of both wheat cultivars BARI Gom 24 and BARI Gom 26 affected by blast infestation indicating that the modulation of nutritional and biochemical contents in wheat grains infected by wheat blast fungus MoT demonstrated in this research is a general phenomenon.

Our result showed that infection of *MoT* significantly reduced the breadth, length, weight, and volume of the wheat grains compared to unaffected healthy grains. Wheat grains that originated from diseased heads had a mix of smaller, lighter, and shriveled grains (Figure 1). Higher infestation rates lowered the grain length, breadth, weight, and volume (Table 1). Similar results were reported by Manandhar et al. (1998) and Urashima et al. (2009). However, severely damaged grains gave appreciable percentage of grain germination. Slightly reduced germination of wheat blast-infected grains were recorded by Urashima et al. (2009) and Gomes et al. (2017). The possible reason may be that the pathogen remains on the outer surface and invades only a few layers of the seed endosperms. The embryo is likely to remain free from the damage by pathogen, and thus, seed germination was not much affected (Urashima et al., 2009).

Grain N, Ca, Mg, and Fe contents significantly increased with increased damage of the grains by the *MoT* infestation is one of the notable findings of our study. On the other hand, P, Cu, Zn, B, and S contents in grains significantly reduced with increase in *MoT* infestation. However, K content was initially increased (up to 40–59% damaged) with an increase in damage, but later on, it was decreased with increase in damage. Jaskulska et al. (2018) reported that blast infestation results in increase in N and C contents in wheat grains. Urashima et al. (2009) also found a similar trend of result for the N content in blast-damaged grains compared to unaffected control. However, our report demonstrated that the severity of grain damage by wheat blast disease is correlated with the increment of grain N and Ca contents. Still, the mechanisms are not clearly understood from the data of this article; however, translocation of N, Ca, and Fe might happen much earlier in the spike than other mineral nutrients. Györi (2017) reported that Cu, Fe, and Zn accumulation are highly dependent on grain N content. In our study, grain Cu, Fe, and Zn contents ranged from 1.20 to 4.20, 53.20 to 89.40, and 12 to 52.20 mg/g, respectively. The exact explanation of such high variability in mineral contents in blast-affected grains are not known. It might be linked with the time of translocation patterns of various mineral nutrients to the gain filling and spike development stages of wheat. In fact, head infection of wheat blast fungus blocks the vascular system in rachis, and thus, nutrients and water cannot move to the growing spike, which results in bleaching of the spike above the point of infection. Several lines of evidence suggest that plant pathogen infection causes variations in mineral nutrient contents in plant products (Uthayakumaran and Wrigley, 2017). Further studies are needed to elucidate the mechanisms of positive (N, Ca, Mg, and Fe) and negative (P, Cu, Zn, B, and S) correlations of mineral nutrient contents in wheat grains with severity of blast infestation.

A strong positive relationship between grain crude protein percentage and level of grain damage by *MoT* infestation was a notable finding in our study. Previously, Martínez et al. (2019) reported that irrespective of the isolates of *Pyricularia oryzae* and cultivars of wheat, blast infestation generally increases the grain protein content. Urashima et al. (2009) also found a higher content of protein in the blast-damaged wheat grains. Miranda et al. (2015) also demonstrated that blast disease changes the physiochemical parameters of the wheat grain. Like *MoT*, infestation by *Meloidogyne graminicola* also increases the grain protein content in wheat (Dimmock and Gooding, 2002). Similarly, Maciel et al. (2014) reported that septoria leaf blotch disease in wheat enhances the grain protein content with an increase in disease severity. A further study is needed to elucidate the underlying mechanisms of the inverse relationship of grain crude protein content with severity of grain damage caused by *MoT*. The moisture, lipid, and ash percentage slightly increased with increased damage caused by *MoT*. High moisture content in grain affects grain yield (Mohan and Gupta, 2015). Grain lipids were degraded endogenously via fungal invasion by oxidation and hydrolysis. An increase in lipid levels in grains is indicative of more utilization of fatty acids by spoilage fungi. However, the reason for increase of ash percentage with the increase in *MoT* infestation is not clear from the data obtained in this study.

We found a significant difference in concentrations of total flavonoid and carotenoids, and antioxidant activities in wheat grains. The grain concentrations of TFC, total carotenoids, and TAA were decreased with increased severity of the wheat blast disease. However, the grain TPC was increased at a certain level of the damage by *MoT* and then decreased with increased infestation. Prevention of oxidative damage, as well as damage caused by pathogen, is controlled by antioxidant activity (Adom et al., 2003; Shahidi and Liyana-Pathirana, 2008). The flavonoids are present in the pericarp and the germ of the wheat grain. About 93% of the total flavonoids in wheat are present in the cell wall-bound form (Adom and Liu, 2002). Carotenoids play important roles as antioxidants, attractants for pollinators, and light-harvesting pigments. The lower the carotenoid level, the lower is the antioxidant capacity, and the lower the protective role against the pathogen. The higher amounts of antioxidant compounds, flavonoids, and carotenoids play a major role in inhibiting fungal attacks as well as provide a barrier to invade in the plant cell (Atanasova-Penichon et al., 2016). The decreasing trends of flavonoids, carotenoids, and antioxidant activities of wheat grains with higher infestation by *MoT* described in this paper have not been reported earlier. Surprisingly, initially, TPC increased with the increase in *MoT* infestation. However, with the infection level at 60% or higher, the amount of phenolic compounds decreased significantly. In wheat grains, a total of 85% phenolic compounds are present in bound form (Kim et al., 2006). The bound phenolic acids and other polyphenols are very important for health benefits (Adom et al., 2003; Kim et al., 2006; Liyana-Pathirana and Shahidi, 2006). Changes in biochemical, minerals, and nutritional components in wheat grains affected by various levels of *MoT* infection and their underlying molecular mechanisms would be helpful for efficient disease management strategies.

## Conclusion

In conclusion, a detailed study of physical, physiological, biochemical, mineral, and nutritional properties of wheat grains affected by varying levels of blast infestation was carried out. The *MoT* infestation significantly increased contents of grain N, crude protein, Ca, Mg, and Fe, but decreased the contents of organic carbon, P, Zn, B, Cu, and S. Concentrations of total flavonoid, and carotenoid and TAA in wheat grains are also remarkably affected by the damage caused by *MoT*. The K content and TPC increased at a certain level of damage; then, they decreased with the increase in *MoT* infestation. Although this study demonstrated significant insights of wheat–*MoT* interactions, further studies are needed to clarify the underlying mechanisms of the modulation of grain minerals, biochemical, nutritional, and antioxidant activities caused by varying levels of *MoT* infestation. Better understanding of the modulation of biochemical and nutritional properties in blast-infested grains may support further decision making and opens a new window for future genetic and functional genomic studies of wheat blast disease.

## Data Availability Statement

All datasets generated for this study are included in the article/Supplementary Material.

## Author Contributions

MS carried out the research, analyzed the data, and wrote the manuscript. DG carried out the research. NM performed the biochemical studies and statistical analysis. PB, MH, and MM collected the field samples. MR quantified nutritional components and their analysis. BM performed the quantification of moisture, ash, and lipid contents in grain and their analysis. TI conceptualized, supervised, and interpreted the data of the research, and wrote and revised the manuscript. All authors read and approved the final manuscript.

## Conflict of Interest

The authors declare that the research was conducted in the absence of any commercial or financial relationships that could be construed as a potential conflict of interest.
