# Supplementary material for: Modulation of Nutritional and Biochemical Properties of Wheat Grains Infected by Blast Fungus Magnaporthe oryzae Triticum Pathotype
Source: Front Microbiol. 2020 Jun 24;11:1174. doi: 10.3389/fmicb.2020.01174 (PMC7344263; doi:10.3389/fmicb.2020.01174)
Supplement: Supplementary file 1 [file Data_Sheet_1.docx]

# **Supplementary** **Table 1: Effects of *MoT* infestation on physical changes and germination percentage of wheat cv. BARI Gom 26**

| Damage Category | Grain Length (mm) | Grain breadth (mm) | % Germination | 1000 grain weight (g) | Volume of 500 grains (mm^3^) |
| --- | --- | --- | --- | --- | --- |
| 1 (No infection) | 7.6 ± 0.08a | 3.2 ± 0.04a | 91.86 ± 0.12a | 45.1 ± 0.31a | 20. 01 ± 0.04a |
| 3 (20-39%) | 7.1 ± 0.05b | 3.13 ± 0.04a | 91.68 ± 0.08a | 42.35 ± 0.03b | 19.52 ± 0.19a |
| 4 (40-59%) | 6.75 ± 0.06c | 2.93 ± 0.2b | 90.67 ± 0.17 b | 40.2 ± 0.20c | 19.50 ± 0.26a |
| CV | 0.12 | 0.12 | 0.45 | 0.74 | 0.64 |

Mean values within a column followed by the same letter do not differ significantly by Fisher’s protected LSD test at (*p* ≤ 0.05). Data are presented as mean ± SE (n = 3).

**Supplementary Table 2: Effects of *MoT* infestation on mineral contents of wheat cv. BARI Gom 26**

| Damage Categories | % N% | % OC | Ca^2+^ (mg/g) | Mg^2+^  (me/100g) | K (me/100g) | P  (μg/g) | Cu  (mg/g) | Fe  (mg/g) | Zn  (mg/g) | B  (μg/g) | S  (μg/g) |
| --- | --- | --- | --- | --- | --- | --- | --- | --- | --- | --- | --- |
| 1 (No infection) | 1.81 ± 0.16b | 3.39 ± 0.30a | 0.20 ± 0.02c | 0.10 ± 0.005a | 1.00 ± 0.005b | 0.28 ± 0.001c | 5.40 ± 0.10a | 41.20 ± 0.69c | 66.10 ± 0.62a | 37.00 ± 0.03a | 0.27 ± 0.005a |
| 3 (20-39%) | 2.27 ± 0.03a | 2.49 ± 0.14b | 0.35 ± 0.02b | 0.11 ± 0.02a | 1.5 ± 0.03a | 0.35 ± 0.02b | 4.20 ± 0.16b | 43.50 ± 0.16b | 54.33 ± 0.62b | 35.0 ± 0.39b | 0.23 ± 0.01b |
| 4 (40-59%) | 2.59 ± 0.01a | 2.00 ± 0.11b | 0.40 ± 0.00a | 0.11 ± 0.00a | 1.5 ± 0.02a | 0.84 ± 0.02a | 4.00 ± 0.03b | 47.10 ± 0.11a | 48.01 ± 0.16c | 31.00± 0.66c | 0.21 ± 0.01b |
| CV | 0.33 | 0.71 | 0.04 | 0.03 | 0.07 | 0.06 | 0.39 | 1.45 | 1.78 | 1.55 | 0.03 |

Mean values within a column followed by the same letter do not differ significantly by Fisher’s protected LSD test at (*p* ≤ 0.05). Data are presented as mean ± SE (n = 3). N, nitrogen; OC, organic carbon; Ca, calcium; Mg, magnesium; K, potassium; P, phosphorus; Cu, copper; Fe, iron; Zn, zinc; B, boron and S, sulfur.

**Supplementary Table 3: Effect of *MoT* infestation on crude protein, moisture, lipid and ash contents of wheat cv. BARI Gom 26**

| Damage Category | Crude protein% | Moisture% | Lipid% | Ash% |
| --- | --- | --- | --- | --- |
| 1 (No infection) | 10.32 ± 0.94b | 17.96 ± 0.62b | 3.39 ± 0.08a | 1.90 ± 0.04a |
| 3 (20-39%) | 12.94 ± 0.17a | 19.67 ± 0.39a | 3.43 ± 0.16a | 1.92 ± 0.17a |
| 4 (40-59%) | 14.76 ± 0.6a | 20.28 ± 0.12a | 3.50 ± 0.09a | 1.95 ± 0.07a |
| CV | 1.91 | 1.50 | 0.40 | 0.37 |

Mean values within a column followed by the same letter do not differ significantly by Fisher’s protected LSD test at (*p* ≤ 0.05). Data are presented as mean ± SE (n = 3).

**Supplementary Table 4: Changes of total flavonoids, total phenolic, total carotenoids concentration and antioxidant activity in variously damaged wheat grains cv. BARI Gom 26 infected by *MoT*.**

| Damage Category | Total flavonoids  (µg quercetin eq./g) | Total phenolic  (µg gallic acid eq./g) | Total Carotenoids (mg Lutein eq./g) | Total antioxidant activity  (µg BHT eq./g) |
| --- | --- | --- | --- | --- |
| 1 (No infection) | 934.91 ± 1.03a | 273.88 ± 0.91c | 5.88 ± 0.30a | 130. 53 ± 0.23a |
| 3 (20-39%) | 637.91 ± 0.49b | 277.88 ± 0.96b | 5.29 ± 0.32a | 75. 14 ± 0.09b |
| 4 (40-59%) | 597.85 ± 0.32c | 400.36 ± 0.62a | 5.11 ± 0.10a | 55.42 ± 0.76c |
| CV | 2.38 | 2.92 | 0.89 | 1.60 |

Mean values within a column followed by the same letter do not differ significantly by Fisher’s protected LSD test at (*p* ≤ 0.05). Data are presented as mean ± SE (n = 3).
